# Erythrocyte indices and localized stage II/III periodontitis in military young men and women: CHIEF oral health study

**DOI:** 10.1186/s12903-022-02455-0

**Published:** 2022-09-17

**Authors:** An-Chieh Feng, Sung-Chiao Tsai, Yen-Po Lin, Kun-Zhe Tsai, Gen-Min Lin

**Affiliations:** 1 Department of Medicine, Hualien Armed Forces General Hospital, No. 163, Jiali Rd., Xincheng Township, Hualien, 97144 Taiwan; 2 Department of Surgery, Tri-Service General Hospital, National Defense Medical Center, Taipei, Taiwan; 3 Department of Stomatology of Periodontology, Mackay Memorial Hospital, Taipei, Taiwan; 4 Departments of Dentistry, Tri-Service General Hospital, Taipei, Taiwan; 5 School of Dentistry, National Defense Medical Center, Taipei, Taiwan; 6 Department of Critical Care Medicine, Taipei Tzu-Chi Hospital, New Taipei City, Taiwan; 7 Department of Internal Medicine, Tri-Service General Hospital, National Defense Medical Center, Taipei, Taiwan

**Keywords:** Erythrocyte indices, Hemoglobin, Localized periodontitis, Mean corpuscular volume, Military young adults

## Abstract

**Background:**

Prior studies have shown an association between generalized periodontitis and anemia in older or undernourished adults. The aim of the study was to examine the associations of erythrocyte indices with localized periodontitis in robust young adults, which has never been reported before.

**Methods:**

The study included 1286 military participants, aged 19–40 years, with regular exercise training in Hualien, Taiwan. Localized periodontitis was grouped to healthy/stage I and stage II/III (n = 803 and 325) in men and (n = 130 and 28) in women according to the 2017 criteria of the world workshop. Systemic inflammation was evaluated by leukocyte counts. Multiple logistic regression analysis with adjustment for age, tobacco smoking status, betel nut chewing status, body mass index and leucocyte counts were used to determine the associations.

**Results:**

Greater mean corpuscular volume in young men [odds ratio (OR) and 95% confidence intervals 1.03 (1.01–1.06)], and greater hematocrit and hemoglobin levels in young women were associated with a higher risk of localized stage II/III periodontitis [OR: 1.17 (1.02–1.34) and 1.60 (1.06–2.41), respectively]. However, there were no associations for erythrocyte counts.

**Conclusions:**

The localized stage II/III periodontitis risk increased with greater erythrocyte indices in robust young adults. This finding could be explained in part by that localized periodontitis may promote physical stress, possibly resulting in an increase of erythrocyte indices. On the other side, greater physical fitness associated with a lower risk of periodontitis may consume iron storage in the body, leading to exercise-induced anemia or smaller erythrocyte volume.

**Supplementary Information:**

The online version contains supplementary material available at 10.1186/s12903-022-02455-0.

## Introduction

Periodontitis related to an immune-inflammatory response to the pathogens in the periodontal tissues [1], affects the supporting tissue of the tooth, and induces the loss of cementum, alveolar bone and periodontal ligament. The prevalence of periodontitis accounts for 45–50% of the world's population and the most severe form influences 11.2%, which is the sixth most common human disease [2]. Dysbiosis of pathogenic microorganisms in the biofilm produce chemokines and proinflammatory cytokines to trigger inflammatory processes in the periodontal tissues [3]. Generalized periodontitis coexisted frequently with other chronic inflammatory diseases, such as metabolic syndrome [4], type 2 diabetes [5], cardiovascular disease [6], chronic obstructive pulmonary disease [7], and anemia [8]. A prior meta-analysis has shown that generalized periodontitis might decrease hemoglobin levels in older ages or undernourished young adults and disturb the balance of iron metabolism, confirming the strength of association between periodontitis and the development tendency of anemia, particularly for severe periodontitis [8].

Anemia is characterized by reduced hemoglobin levels or numbers of red blood cells (erythrocytes) and affects a third of the world's population [9]. Half of the cases are due to iron deficiency [10]. Experimental models suggested that iron sequestration is part of a natural defense against invading pathogens [11]. An effective immune system is depriving the stored iron, a vital nutrient for the proliferation of invading pathogens [12]. To our knowledge, in nourished young adults, iron storages are abundant to effectively provide hemoglobin synthesis, which is commonly greater than 13.0 g/dL for men and 11.0 g/dL for women [13], except those who has a thalassemia minor trait. In addition, generalized chronic periodontitis was rarely found in nourished young adults lower than 40 years, while localized periodontitis was present in more than 80% of the robust young adults in our prior studies [3]. Greater body mass index (BMI) has been well established as a risk factor of periodontitis in the general population of any ages, whereas an association with higher hemoglobin levels was particularly present in the youth and women [14, 15]. Localized periodontitis begins inflammation process from young ages, and progresses to generalized chronic periodontitis at later life. BMI and systemic inflammation levels may play a role for erythrocyte indices in young adults. However, the association of localized periodontitis with erythrocyte indices, e.g., hemoglobin levels in robust young adults has not been investigated before. Therefore, the purpose of this study was to clarify the relationships in military young subjects.

## Materials and methods

### Study population

This cross-sectional study population was obtained from the ancillary study of the cardiorespiratory fitness and hospitalization events in armed forces (CHIEF oral health) study [3]. The ancillary CHIEF-Oral Health study was carried out at the Hualien Armed Forces General Hospital in Hualien, Taiwan during 2018–2020, including 1389 young military adults. The exclusion criteria in this study included women who were pregnant or within 14 days after menstruation and those who had type 1 diabetes, active cancer, history of seizure with anticonvulsant therapy, fewer than 16 teeth, missing relevant data, treated and well-maintained periodontitis, or generalized periodontitis. These participants received the annual military health examination, for oral health, physical and laboratory examinations. Demographic variables were obtained from a personal questionnaire (service specialty, education level, betel nut consumption status, and smoking status). The level of education was categorized by the highest school grade (senior high school, college or university, and postgraduate degree). Betel nut chewing status and smoking status, both of which have been identified as crucial risk factors of periodontal diseases [16], were categorized into never/former and current.

### Clinical and demographic measures

Before the health examination, participants were required to fast for 12 h. Anthropometric measurements for waist circumference, body height, and body weight were performed in a standing position. BMI was defined as body weight divided by square of body height (kg/m^2^). Hemodynamic parameters of each participant, i.e. systolic blood pressure (SBP) and diastolic blood pressure (DBP) were measured at rest for 15 min in a sitting position using the FT-201 automated monitoring machine (Parama-Tech Co Ltd, Fukuoka, Japan) [17]. The serum biochemical concentrations and hematological indices were measured by the auto analyzer (Olympus AU640 auto analyzer (Olympus, Kobe, Japan) and the XT-2000-I automated hematology analyzer (Sysmex Corporation, Kobe, Japan), respectively [18–20]. Systemic inflammation was assessed by leucocyte counts [21].

### Periodontal status measures

Full mouth periodontal charting was recorded at six sites of each tooth, including probing pocket depth (PPD), clinical attachment loss (CAL) and dichotomous bleeding on probing (BoP). In addition, data of furcation involvement, tooth mobility, remaining teeth numbers, full mouth bleeding scores and full mouth radiographic images were collected. Based on the 2017 world workshop defined criteria of the American Academy of Periodontology and the European Federation of Periodontology [22], initial periodontitis (stage I) was defined as (1) interdental CAL between 1 and 2 mm at the side of greatest loss, or (2) radiographic bone loss < 15%, or (3) no tooth loss due to periodontitis, or (4) maximum PPD ≤ 4 mm, or mostly horizontal bone loss; Moderate (stage II) periodontitis was defined as (1) interdental CAL between 3 and 4 mm at the side of greatest loss, or (2) radiographic bone loss between 15 and 33%, or (3) no tooth loss due to periodontitis, or (4) maximum PPD ≤ 5 mm, or mostly horizontal bone loss; and severe (stage III) periodontitis was defined as (1) interdental CAL ≥ 5 mm at the side of greatest loss, or (2) radiographic bone loss extending to mid-third of root and beyond, or (3) with tooth loss due to periodontitis, or (4) in addition to moderate periodontitis severity further with PPD ≥ 6 mm, or vertical bone loss ≥ 3 mm, or class II/III furcation involvement, or moderate ridge defect. Before the beginning of this study, four dentists were trained for the standardization of procedure in consecutive 100 young patients. Discrepancies between two examiners were discussed by consensus. In the last month of the warm-up period, the consistencies for the staging and grading of periodontitis among the examiners were both greater than 90%. After the study begun, the dental examinations were initially performed by Dr. Tsai, Kun-Zhe for the grading of periodontitis. A follow-up examination and treatment for the oral pathologies of each participant was performed in detail by other dentists in the Outpatient Department in 1 month, and the inter-observer agreement (kappa coefficient) for verifying the stage of periodontitis was estimated 90.6%. As the study was aimed at physically active adults, the extent of periodontitis was merely restricted to the localized (< 30% of teeth involved) [22]. This study design and protocol were reviewed and approved by the human ethics board of the Mennonite Christian Hospital (No. 16-05-008) in Hualien City, Taiwan and written informed consents were obtained from all participants. Also, this study was performed in accordance with the Helsinki Declaration of 1975, as revised in 2013.

### Statistical analysis

A sample size of 1258 or more subjects is sufficient to have a confidence level of 99% that the real value is within ± 1% of the measured value. Since the 2017 world workshop for the goal of periodontitis managements was to downgrade the greater stage II–IV to the healthy or stage I with a stable oral condition [22], we classified our subjects into two groups: the healthy/stage I and the stage II/III periodontitis. The clinical features were shown as mean ± standard deviation (SD) for continuous data, and numbers (%) for categorical data. The continuous variables were compared by one-way analysis of variance (ANOVA), and the categorical variables were compared by chi-square test. Smooth curve fitting analysis was performed for displaying spline smoothing plots to show whether there were non-linear relationships of erythrocyte indices with the risk of stage II/III periodontitis (log_e_ transformation for the ratio of stage II/III periodontitis to the healthy with an adjustment for age based on sex. If there was a non-linear relationship, the likelihood ratio test was used to compare the difference using a segmented regression model greater and lower than the turning point in men and women [3]. Sex-specific multiple logistic regression analysis was utilized to determine the odds ratio (OR) and 95% confidence intervals (CI) of erythrocyte indices for localized stage II/III periodontitis. The covariates were adjusted in two models for severer periodontitis. In model 1, age, educational levels, smoking, and betel-nut chewing, and BMI were adjusted. In model 2, the leukocyte counts were further adjusted. In addition, we also provided the results using different group classifications in which the stage I periodontitis was treated as an independent exposure variable in Additional file 1: Tables S1 and S2. SPSS statistical software was used for all the statistical analyses (IBM Corp. Released 2017. IBM SPSS Statistics for Windows, Version 25.0. Armonk, NY: IBM Corp.).

## Results

Of the 1389 young military participants in the initial enrollment, 103 subjects were excluded due to the presence of pregnancy (N = 8), lower than 2 weeks after menstruation (N = 74), type 1 diabetes (N = 1), active cancer on chemotherapy (N = 1), seizure with anticonvulsants (N = 1), fewer than 16 teeth (N = 3), missing relevant data (N = 12), treated and well-maintained periodontitis (N = 1), and generalized periodontitis (N = 2). Finally, a sample of 1286 military subjects of 1128 males and 158 females, aged 19–40 years were included for final analysis.

Table 1 shows the clinical characteristics of men (1128) and women (N = 158), subdivided into those with healthy periodontal status and localized stage II/III periodontitis. In men, those with localized stage II/III periodontitis (N = 325) were older in age, had a greater prevalence of metabolic syndrome and active betel-nut chewing, and greater BP, waist circumference, BMI, total cholesterol, serum triglycerides, leucocyte counts, mean corpuscular volume (MCV) and full mouth bleeding score. In women, those with localized stage II/III periodontitis (N = 28) had greater waist circumference, serum hematocrit, serum hemoglobin and bleeding score, and lower high-density lipoprotein.

**Table 1:** Clinical characteristics of the healthy/ localized stage I periodontitis and the localized stage II/III periodontitis

Characteristics	Male (N = 1128)			Female (N = 158)		
	Healthy/localized stage I periodontitis (N = 803)	Localized stage II/III periodontitis (N = 325)	*p* value	Healthy/localized stage I periodontitis (N = 130)	Localized stage II/III periodontitis (N = 28)	*p* value
Age (years old)	30.10 ± 5.83	31.91 ± 5.54	**< 0.001**	27.97 ± 6.25	26.82 ± 6.37	0.38
Education level						
Up to senior high school	238 [29.6]	78 [24.0]	0.15	65 [50.0]	19 [67.9]	0.21
College/University degree	542 [67.5]	238 [73.2]		64 [49.2]	9 [32.1]	
Postgraduate degree	23 [2.9]	9 [2.8]		1 [0.8]	0 [0.0]	
Metabolic syndrome	464 [57.8]	217 [66.8]	**0.005**	34 [26.2]	11 [39.3]	0.16
Unhealthy behavior						
Current betel nut chewer	41 [5.1]	33 [10.2]	**0.002**	1 [0.8]	1 [3.6]	0.22
Current tobacco smoker	160 [19.9]	73 [22.5]	0.33	3 [2.3]	2 [7.1]	0.18
Systolic blood pressure (mm Hg)	122.56 ± 11.32	124.40 ± 13.31	**0.01**	111.67 ± 11.62	110.46 ± 10.52	0.61
Diastolic blood pressure (mm Hg)	74.32 ± 9.46	75.69 ± 11.59	**0.03**	67.95 ± 9.44	68.11 ± 8.69	0.93
Waist circumference (cm)	85.36 ± 10.32	88.80 ± 10.67	**< 0.001**	75.99 ± 10.00	80.12 ± 8.36	**0.03**
Body mass index (kg/m^2^)	25.69 ± 3.70	26.84 ± 3.65	**< 0.001**	23.36 ± 3.42	24.25 ± 4.13	0.23
Blood test						
Total cholesterol (mg/dL)	180.55 ± 34.74	188.27 ± 36.64	**0.001**	174.26 ± 34.62	179.68 ± 30.34	0.44
HDL-C (mmol/L)	48.17 ± 9.99	47.28 ± 10.44	0.18	58.46 ± 11.73	53.39 ± 10.34	**0.03**
LDL-C (mmol/L)	110.94 ± 31.29	114.33 ± 31.04	0.09	98.24 ± 31.05	107.54 ± 27.43	0.14
Serum triglycerides (mg/dL)	127.06 ± 99.78	162.34 ± 129.21	**< 0.001**	79.55 ± 40.70	93.18 ± 39.36	0.10
Fasting glucose (mg/dL)	93.65 ± 16.41	93.74 ± 16.41	0.93	88.73 ± 8.60	86.61 ± 9.23	0.24
Platelet count (10^3^/uL)	252.89 ± 55.26	254.05 ± 53.77	0.74	271.91 ± 57.14	289.64 ± 60.34	0.14
Leucocyte count (10^3^/uL)	6.98 ± 1.66	7.26 ± 1.75	**0.01**	6.77 ± 1.87	7.24 ± 1.82	0.23
Erythrocyte count (10^3^/uL)	5.34 ± 0.43	5.31 ± 0.40	0.28	4.66 ± 0.45	4.76 ± 0.37	0.27
HCT (%)	46.26 ± 2.57	46.62 ± 2.66	**0.03**	40.27 ± 3.04	41.80 ± 3.66	**0.02**
MCV (fL)	86.86 ± 6.28	88.04 ± 5.99	**0.004**	86.83 ± 7.60	87.85 ± 5.85	0.50
Hemoglobin (g/dL)	15.52 ± 0.96	15.59 ± 0.94	0.27	13.19 ± 1.11	13.73 ± 1.15	**0.02**
Range: (min–max)	10.7–18.3	13.0–18.5		10.2–15.6	11.5–15.7	
Full mouth bleeding scores	4.03 ± 1.72	17.77 ± 3.38	**< 0.001**	4.22 ± 1.78	16.75 ± 3.37	**< 0.001**

A *p*-value <0.05 was defined as statistically significant and highlighted in bold

Continuous variables are expressed as mean ± standard deviation, and categorical variables as N [%]

*HDL-C* high-density lipoprotein cholesterol, *LDL-C* low-density lipoprotein cholesterol, *HCT* hematocrit, *MCV* mean corpuscular volume

**Fig. 1:**
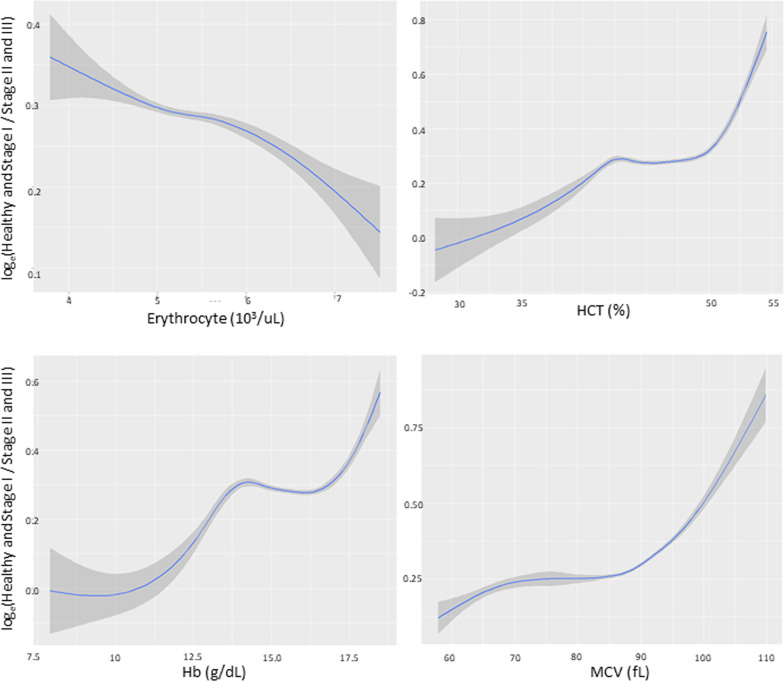
In men, a positive linear relationship curve was noted in hematocrit (HCT), hemoglobin (Hb) and mean corpuscular volume (MCV) (x-axis) against the stage II/III localized periodontitis risk (y-axis). In addition, the risk of stage II/III periodontitis was not increased within a range of HCT (42.5–50.0%) and Hb (13.5–16.0 g/dL)

**Fig. 2:**
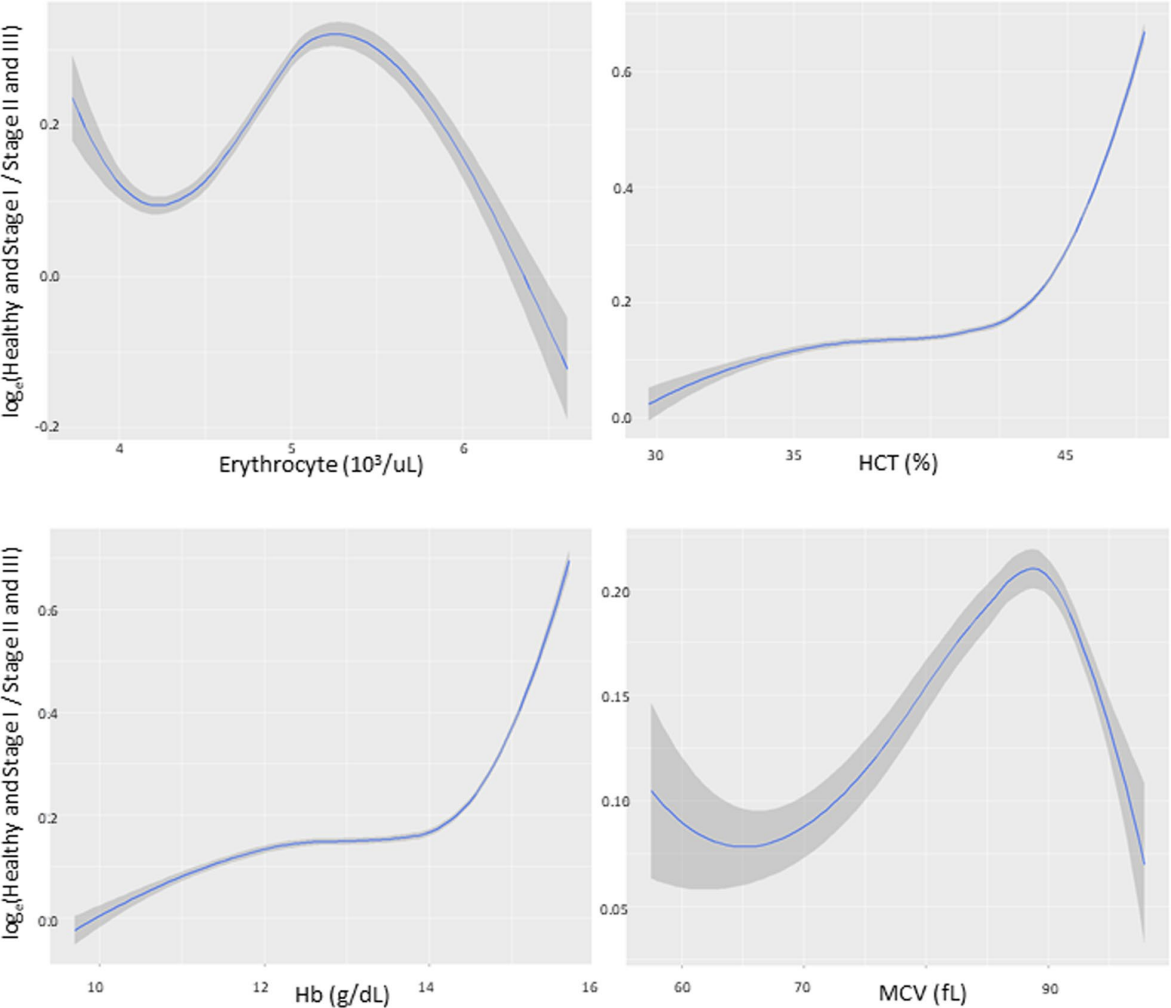
In women, a positive linear relationship curve was observed in hematocrit (HCT), hemoglobin (Hb) and mean corpuscular volume (MCV) (x-axis) against the risk of stage II/III localized periodontitis (y-axis); however an inverse linear relationship curve was observed in erythrocyte counts (x-axis) in both men and women

Figures 1 and 2 present the results of spline smoothing plots respectively for levels of erythrocyte counts, hematocrit, MCV, and hemoglobin against the risk of localized stage II/III periodontitis in young men and women. Of these, a positive linear relationship curve was observed in hematocrit, hemoglobin, and MCV. However, the localized stage II/III periodontitis risk was not increased within a range of hematocrit (42.5–50.0%) and hemoglobin (13.5–16.0 g/dL) (a flash sign) in men but not in women. In contrast, an inverse linear relationship curve was observed in erythrocyte counts in both men and women.

Table 2 demonstrates the multiple logistic regression results for the associations between the erythrocyte indices and localized stage II/III periodontitis. In men, greater MCV was associated with higher localized stage II/III periodontitis risk in model 1 [OR: 1.03, 95% CI 1.01–1.06] and in model 2 (OR: 1.03, 95% CI 1.01–1.06). However, there were no associations for erythrocyte counts, hematocrit, and hemoglobin. In women, the association of hematocrit with localized stage II/III periodontitis was significantly presented in model 1 and model 2 (OR: 1.17, 95% CI 1.02–1.34 and 1.17, 95%CI 1.02–1.34, respectively). In addition, hemoglobin was also associated with the localized stage II/III periodontitis risk in model 1 and model 2 (OR: 1.60, 95% CI 1.06–2.42 and 1.60, 95%CI 1.06–2.41, respectively).

**Table 2:** Multiple logistic regression analysis for localized stage II/III periodontitis with various erythrocyte indices

	Model 1			Model 2		
	OR	95% CI	*p* value	OR	95% CI	*p* value
*Male*						
Erythrocyte (10^3^/uL)	0.79	0.57–1.09	0.14	0.77	0.56–1.06	0.11
HCT (%)	1.05	1.02–1.07	0.08	1.04	0.99–1.10	0.13
MCV (fL)	1.03	1.01–1.06	**0.005**	1.03	1.01–1.06	**0.006**
Hemoglobin (g/dL)	1.05	0.92–1.21	0.45	1.04	0.90–1.19	0.59
*Female*						
Erythrocyte (10^3^/uL)	1.63	0.66–4.04	0.29	1.61	0.65–3.98	0.30
HCT (%)	1.17	1.02–1.34	**0.02**	1.17	1.02–1.34	**0.02**
MCV (fL)	1.03	0.96–1.10	0.45	1.03	0.96–1.09	0.45
Hemoglobin (g/dL)	1.60	1.06–2.42	**0.02**	1.60	1.06–2.41	**0.02**

A *p*-value <0.05 was defined as statistically significant and highlighted in bold

Data are presented as odds ratios and 95% confidence intervals (CI) using multiple logistic regression analysis for

Model 1: age, educational levels, tobacco smoking, betel nut chewing and body mass index adjustments

Model 2: age, educational levels, tobacco smoking, betel nut chewing, body mass index and leucocyte adjustments

Abbreviations: CI, confidence interval; HCT, hematocrit; MCV, mean corpuscular volume; OR, odds ratio

## Discussion

This is the first study to demonstrate associations of erythrocyte indices with localized stage II/III periodontitis in robust young adults. There were 28.8% of young men and 17.7% of young women found to have localized stage II/III periodontitis among military personnel in Taiwan. Interestingly, the present study revealed an inverse association between hemoglobin levels and the presence of localized periodontitis. Different from prior studies, which reported an association between anemia and generalized periodontitis, the present study was carried out on a unique group of robust military adults.

There have been four studies done on relatively young adults, aged 20–55 years, in India, which reported an association between generalized periodontitis and anemia that was contrary to the present study findings [23–26]. The inconsistent conclusion from the India studies might result from two aspects: (1) the young adults they included were with malnutrition status and low physical activity; (2) the young adults they included were with generalized chronic periodontitis. The mean hemoglobin of control group was 12.6 g/dL (n = 30 males) and 13.3 g/dL (n = 27 males; n = 23 females) in the India studies done by Anumolu et al. and Gokhale et al. [23, 26], repsecitvely, which were lower than the suggested normal reference for young nourished adults, e.g., the mean hemoglobin in our male control group (15.5 g/dL) and female control group (13.2 g/dL) in the present study. General nutrition status reflects systemic anti-inflammatory ability and under-nourished adults can be less tolerable to infection.

Second, periodontitis is considered as an inflammatory disease from localized early stage to generalized chronic stage. Though it was not fully understood, the pathogenic processes of anemia are considered to be mediated through the actions of tumor necrosis factor (TNF)-α and interleukins (IL)-1 and -6, and interferon [27]. Periodontitis is predominantly caused by subgingival oral biofilm which can activate pro-inflammatory cytokines by endotoxins release [3]. Pro-inflammatory cytokines, in particular IL-1, IL-6 and TNF-α might down-regulate the erythropoiesis in the bone marrow and result in lower numbers of erythrocytes and consequently lower hemoglobin levels [28, 29], particularly in the iron-deficient state of the elderly. Furthermore, the pro-inflammatory cytokines have also been reported to facilitate the secretion of ferritin [30]. Ferritin, as an iron-binding protein, plays an important role in iron storage and recycling and declines the hemoglobin concentrations [8]. Greater stage of periodontitis in the elderly usually accompanied with various degrees of bone loss had a higher possibility of developing anemia of inflammation [8, 29, 31], a hallmark of chronic and persistent inflammatory diseases with prolonged immune activation [32], is found by insufficient erythropoietin productions and decreased response of erythroid progenitors to erythropoietin [33]. In summary, along with periodontal disease progression to generalized periodontitis, iron storage in the body might be consumed rapidly, thereby presenting an anemic status, even in young adults in the India studies [23–26].

In the present study, the erythrocyte indices were found positively correlated with localized staged II/III periodontitis among robust young adults. These findings may be explained in part by that high cardiorespiratory fitness levels have been associated with a lower risk of periodontitis in young adults [34]. However, it was also well known that high cardiorespiratory fitness levels have been associated with iron consumption, leading to exercise-induced anemia [35, 36]. Since women were more predisposed to iron-deficiency anemia than men, which might lead to a sex difference in the presentations of erythrocyte indices that women were more likely to have a lower hematocrit and hemoglobin level. On the other respect, those young adults with localized periodontitis and greater BMI in the present study might experience higher physical stress while on regular exercise training, which has been observed with an increase of erythrocyte indices in an animal model [37]. Based on these evidence mentioned above, we acknowledged that the relationships between erythrocyte indices and periodontitis should be interpreted carefully, which might vary by the stage of periodontitis, BMI and levels of physical activity or fitness of the study population.

In the present study, there were some limitations. Firstly, it was impossible to exclude residual confounders that may lead to a bias. For instance, response bias in self-report measures might exist since personal considerations to being good in the military personnel. Second, this study had a cross-sectional design, so that the temporality and causality could not be assessed. Third, there were several confounders to affect erythrocyte indices in young women because of their menstrual cycle-related blood loss despite that the blood sample drawing time was unified. Fourth, although we have adjusted for BMI and leukocyte counts in the logistic models, the effect of physical fitness on periodontitis could not be adequately controlled for explaining the findings. In contrast, there were several strengths in the present study. First, this was the first large study for robust young adults investigating the association between erythrocyte indices and localized stage II/III periodontitis. Second, our study had a large sample size that could provide sufficient power to make gender-specific associations between erythrocyte indices and periodontitis.

## Conclusion

The localized stage II/III periodontitis risk increased with greater erythrocyte indices in robust young adults. This finding could be explained in part by that localized periodontitis and greater BMI may promote physical stress, possibly resulting in an increase of erythrocyte indices. On the other end, in young adults, greater physical fitness associated with a lower risk of periodontitis may consume iron storage in the body, leading to exercise-induced anemia or smaller erythrocyte volume. The clinical relevance in the study highlights that the presence of localized periodontitis in robust young adults reflects lower levels of erythrocyte indices, e.g., anemia, and was contrary to the previous studies for generalized periodontitis and anemia in older individuals.

## Supplementary Information

**Additional file 1. Table S1**. Clinical Characteristics of the Healthy, the Localized Stage I Periodontitis and the Localized Stage II/III Periodontitis. **Table S2**. Multiple Logistic Regression Analyses for Localized Stage I and Stage II/III Periodontitis with Erythrocyte Indices.

## Data Availability

The datasets generated and/or analysed during the current study are not publicly available due to materials obtained from the military in Taiwan, which were confidential, but are available from the corresponding author on reasonable request.

## References

[1] Cekici A, Kantarci A, Hasturk H, Van Dyke TE (2014). Inflammatory and immune pathways in the pathogenesis of periodontal disease. Periodontol 2000.

[2] Kassebaum NJ, Bernabé E, Dahiya M, Bhandari B, Murray CJ, Marcenes W (2014). Global burden of severe periodontitis in 1990–2010: a systematic review and meta-regression. J Dent Res.

[3] Tsai KZ, Huang RY, Cheng WC, Su FY, Lin YP, Chang CY, Lin GM (2021). Comparisons of various anthropometric indexes with localized stage II/III periodontitis in young adults: the CHIEF oral health study. J Periodontol.

[4] Jepsen S, Suvan J, Deschner J (2020). The association of periodontal diseases with metabolic syndrome and obesity. Periodontol 2000.

[5] Preshaw PM, Alba AL, Herrera D, Jepsen S, Konstantinidis A, Makrilakis K, Taylor R (2012). Periodontitis and diabetes: a two-way relationship. Diabetologia.

[6] Sanz M, Marco Del Castillo A, Jepsen S, Gonzalez-Juanatey JR, D'Aiuto F, Bouchard P, Chapple I, Dietrich T, Gotsman I, Graziani F, Herrera D, Loos B, Madianos P, Michel JB, Perel P, Pieske B, Shapira L, Shechter M, Tonetti M, Vlachopoulos C, Wimmer G (2020). Periodontitis and cardiovascular diseases: consensus report. J Clin Periodontol.

[7] Martos R, Márton I (2011). Possible correlations between periodontitis and chronic obstructive pulmonary disease. Review of the literature. Fogorv Sz.

[8] Wu D, Lin Z, Zhang S, Cao F, Liang D, Zhou X (2020). Decreased hemoglobin concentration and iron metabolism disorder in periodontitis: systematic review and meta-analysis. Front Physiol.

[9] Kassebaum NJ, Jasrasaria R, Naghavi M, Wulf SK, Johns N, Lozano R, Regan M, Weatherall D, Chou DP, Eisele TP, Flaxman SR, Pullan RL, Brooker SJ, Murray CJ (2014). A systematic analysis of global anemia burden from 1990 to 2010. Blood.

[10] Kassebaum NJ, GBD 2013 Anemia Collaborators (2016). The global burden of anemia. Hematol Oncol Clin N Am.

[11] Fraenkel PG (2017). Anemia of inflammation: a review. Med Clin N Am.

[12] Poggiali E, Migone De Amicis M, Motta I (2014). Anemia of chronic disease: a unique defect of iron recycling for many different chronic diseases. Eur J Intern Med.

[13] Fulgoni VL, Agarwal S, Kellogg MD, Lieberman HR (2019). Establishing pediatric and adult RBC reference intervals with NHANES data using piecewise regression. Am J Clin Pathol.

[14] Jeong HR, Lee HS, Shim YS, Hwang JS (2022). Positive associations between body mass index and hematological parameters, including RBCs, WBCs, and platelet counts, in Korean children and adolescents. Children (Basel).

[15] Qin Y, Melse-Boonstra A, Pan X, Yuan B, Dai Y, Zhao J, Zimmermann MB, Kok FJ, Zhou M, Shi Z (2013). Anemia in relation to body mass index and waist circumference among Chinese women. Nutr J.

[16] Giovannoni ML, Valdivia-Gandur I, Lozano de Luaces V, Varela Véliz H, Balasubbaiah Y, Chimenos-Küstner E (2018). Betel and tobacco chewing habit and its relation to risk factors for periodontal disease. Oral Dis.

[17] Lin GM, Li YH, Lee CJ, Shiang JC, Lin KH, Chen KW, Chen YJ, Wu CF, Lin BS, Yu YS, Lin F, Su FY, Wang CH (2016). Rationale and design of the cardiorespiratory fitness and hospitalization events in armed forces study in Eastern Taiwan. World J Cardiol.

[18] Lin YK, Liu PY, Fan CH, Tsai KZ, Lin YP, Lee JM, Lee JT, Lin GM (2020). Metabolic biomarkers and long-term blood pressure variability in military young male adults. World J Clin Cases.

[19] Lin YP, Tsai KZ, Chang CY, Su FY, Han CL, Lin GM (2021). Tobacco smoking and association between betel nut chewing and metabolic abnormalities among military males: the CHIEF study. Endocr Metab Immune Disord Drug Targets.

[20] Lin JW, Tsai KZ, Chen KW, Su FY, Li YH, Lin YP, Han CL, Lin F, Lin YK, Hsieh CB, Lin GM (2019). Sex-specific association between serum uric acid and elevated alanine aminotransferase in a military cohort: the CHIEF study. Endocr Metab Immune Disord Drug Targets.

[21] Tonetti MS, Greenwell H, Kornman KS (2018). Staging and grading of periodontitis: Framework and proposal of a new classification and case definition. J Periodontol.

[22] Chung PS, Tsai KZ, Lin YP, Lin YK, Lin GM (2020). Association between leukocyte counts and physical fitness in male military members: the CHIEF study. Sci Rep.

[23] Gokhale SR, Sumanth S, Padhye AM (2010). Evaluation of blood parameters in patients with chronic periodontitis for signs of anemia. J Periodontol.

[24] Patel MD, Shakir QJ, Shetty A (2014). Interrelationship between chronic periodontitis and anemia: a 6-month follow-up study. J Indian Soc Periodontol.

[25] Khan NS, Luke R, Soman RR, Krishna PM, Safar IP, Swaminathan SK (2015). Qualitative assessment of red blood cell parameters for signs of anemia in patients with chronic periodontitis. J Int Soc Prev Community Dent.

[26] Anumolu VN, Srikanth A, Paidi K (2016). Evaluation of the relation between anemia and periodontitis by estimation of blood parameters: a cross-sectional study. J Indian Soc Periodontol.

[27] Županić-Krmek, Sučić M, Bekić D (2014). Anemia of chronic disease: illness or adaptive mechanism. Acta Clin Croat.

[28] Vreugdenhil G, Löwenberg B, Van Eijk HG, Swaak AJ (1992). Tumor necrosis factor alpha is associated with disease activity and the degree of anemia in patients with rheumatoid arthritis. Eur J Clin Investig.

[29] Hutter JW, van der Velden U, Varoufaki A, Huffels RA, Hoek FJ, Loos BG (2001). Lower numbers of erythrocytes and lower levels of hemoglobin in periodontitis patients compared to control subjects. J Clin Periodontol.

[30] Huang W, Zhan Y, Zheng Y, Han Y, Hu W, Hou J (2019). Up-regulated ferritin in periodontitis promotes inflammatory cytokine expression in human periodontal ligament cells through transferrin receptor via ERK/P38 MAPK pathways. Clin Sci (Lond).

[31] Botelho J, Machado V, Hussain SB, Zehra SA, Proença L, Orlandi M, Mendes JJ, D'Aiuto F (2021). Periodontitis and circulating blood cell profiles: a systematic review and meta-analysis. Exp Hematol.

[32] Weiss G, Ganz T, Goodnough LT (2019). Anemia of inflammation. Blood.

[33] Spoto B, Kakkar R, Lo L, Devalaraja M, Pizzini P, Torino C, Leonardis D, Cutrupi S, Tripepi G, Mallamaci F, Zoccali C (2019). Serum erythroferrone levels associate with mortality and cardiovascular events in hemodialysis and in CKD patients: a two cohorts study. J Clin Med.

[34] Ferreira RO, Corrêa MG, Magno MB, Almeida APCPSC, Fagundes NCF, Rosing CK, Maia LC, Lima RR (2019). Physical activity reduces the prevalence of periodontal disease: systematic review and meta-analysis. Front Physiol.

[35] Tsai KZ, Lai SW, Hsieh CJ, Lin CS, Lin YP, Tsai SC, Chung PS, Lin YK, Lin TC, Ho CL, Han CL, Kwon Y, Hsieh CB, Lin GM (2019). Association between mild anemia and physical fitness in a military male cohort: the CHIEF study. Sci Rep.

[36] Lai SW, Tsai KZ, Lin YP, Liu PY, Lin YK, Chang PY, Dai MS, Chao TY, Han CL, Lin GM (2021). Association of red blood cell size and physical fitness in a military male cohort: the CHIEF study. Scand J Med Sci Sports.

[37] Caporossi LS, Rosa da Silva A, Delle V, Semenoff TA, Miranda-Pedro FL, Borges ÁH, Segundo Semenoff-Segundo A (2010). Effect of two models of stress associated with ligature-induced periodontitis on hematological parameters in rats. Revista Odonto Ciência.

