# Supplementary material for: Erythrocyte indices and localized stage II/III periodontitis in military young men and women: CHIEF oral health study
Source: BMC Oral Health. 2022 Sep 17;22:404. doi: 10.1186/s12903-022-02455-0 (PMC9482174; doi:10.1186/s12903-022-02455-0)
Supplement: Supplementary file 1 — Additional file 1. Table S1. Clinical Characteristics of the Healthy, the Localized Stage I Periodontitis and the Localized Stage II/III Periodontitis. Table S2. Multiple Logistic Regression Analyses for Localized Stage I and Stage II/III Periodontitis with Erythrocyte Indices. [file 12903_2022_2455_MOESM1_ESM.docx]

ADDITIONAL FILE 1: TABLE S1 Clinical Characteristics of the Healthy, the Localized Stage I Periodontitis and the Localized Stage II/III Periodontitis

|  | Male  (N =1,128) | | | |  | Female  (N =158) | | | |
| --- | --- | --- | --- | --- | --- | --- | --- | --- | --- |
| Characteristics | Healthy  (N =670) | Stage I  (N =133) | Stage II/III  (N =325) | p-value |  | Healthy  (N =115) | Stage I  (N =15) | Stage II/III  (N =28) | p-value |
| Age (years old) | 30.55 ± 5.67 | 27.87 ± 6.13 | 31.91 ± 5.54 | <0.001 |  | 28.43 ± 6.22 | 24.47 ± 5.50 | 26.82 ± 6.37 | 0.04 |
| Education level |  |  |  |  |  |  |  |  |  |
| Up to senior high school | 176 [26.3] | 62 [46.6] | 78 [24.0] | <0.001 |  | 55 [47.8] | 10 [66.7] | 19 [67.9] | 0.28 |
| College/University degree | 474 [70.7] | 68 [51.1] | 238 [73.2] |  |  | 59 [51.3] | 5 [33.3] | 9 [32.1] |  |
| Postgraduate degree | 20 [3.0] | 3 [2.3] | 9 [2.8] |  |  | 1 [0.9] | 0 [0.0] | 0 [0.0] |  |
| Metabolic syndrome | 393 [58.7] | 71 [53.4] | 217 [66.8] | 0.01 |  | 31 [27.0] | 3 [20.0] | 11 [39.3] | 0.32 |
| Unhealthy behavior |  |  |  |  |  |  |  |  |  |
| Current betel nut chewer | 32 [4.8] | 9 [6.8] | 33 [10.2] | 0.006 |  | 0 [0.0] | 1 [6.7] | 1 [3.6] | 0.04 |
| Current tobacco smoker | 122 [18.2] | 38 [28.6] | 73 [22.5] | 0.01 |  | 1 [0.9] | 2 [13.3] | 2 [7.1] | 0.01 |
| Systolic blood pressure (mm Hg) | 123.15 ± 11.28 | 119.55 ± 11.07 | 124.40 ± 13.31 | <0.001 |  | 111.93 ± 11.41 | 109.67 ± 13.36 | 110.46 ± 10.52 | 0.68 |
| Diastolic blood pressure (mm Hg) | 75.16 ± 9.46 | 70.05 ± 8.29 | 75.69 ± 11.59 | <0.001 |  | 68.30 ± 9.53 | 65.27 ± 8.49 | 68.11 ± 8.69 | 0.49 |
| Waist circumference (cm) | 85.35 ± 10.55 | 85.42 ± 9.11 | 88.80 ± 10.67 | <0.001 |  | 76.17 ± 10.01 | 73.66 ± 9.96 | 80.12 ± 8.36 | 0.07 |
| Body mass index (kg/m^2^) | 25.63 ± 3.66 | 25.99 ± 3.89 | 26.84 ± 3.65 | <0.001 |  | 23.37 ± 3.30 | 23.29 ± 4.34 | 24.25 ± 4.13 | 0.48 |
| Blood test |  |  |  |  |  |  |  |  |  |
| Total cholesterol (mg/dL) | 182.06 ± 35.16 | 172.93 ± 31.57 | 188.27 ± 36.64 | <0.001 |  | 173.92 ± 27.80 | 176.87 ± 68.88 | 179.68 ± 30.34 | 0.71 |
| HDL-C (mmol/L) | 48.78 ± 10.11 | 45.13 ± 8.78 | 47.28 ± 10.44 | <0.001 |  | 58.61 ± 11.43 | 57.33 ± 14.28 | 53.39 ± 10.34 | 0.10 |
| LDL-C (mmol/L) | 112.05 ± 31.69 | 105.32 ± 28.59 | 114.33 ± 31.04 | 0.01 |  | 97.82 ± 24.60 | 101.47 ± 62.81 | 107.54 ± 27.43 | 0.31 |
| Serum triglycerides (mg/dL) | 126.06 ± 100.66 | 132.11 ± 95.40 | 162.34 ± 129.21 | <0.001 |  | 79.84 ± 41.53 | 77.27 ± 34.79 | 93.18 ± 39.36 | 0.26 |
| Fasting glucose (mg/dL) | 92.68 ± 14.78 | 98.56 ± 22.38 | 93.74 ± 16.41 | 0.001 |  | 88.17 ± 7.29 | 93.00 ± 15.03 | 86.61 ± 9.23 | 0.066 |
| Platelet count (10^3^/uL) | 253.60 ± 56.47 | 249.34 ± 47.20 | 254.05 ± 53.77 | 0.67 |  | 270.18 ± 57.33 | 285.13 ± 55.75 | 289.64 ± 60.34 | 0.21 |
| Leucocyte count (10^3^/uL) | 6.97 ± 1.67 | 7.04 ± 1.59 | 7.26 ± 1.75 | 0.03 |  | 6.79 ± 1.88 | 6.56 ± 1.82 | 7.24 ± 1.82 | 0.43 |
| Erythrocyte count (10^3^/uL) | 5.36 ± 0.44 | 5.28 ± 0.37 | 5.31 ± 0.40 | 0.08 |  | 4.68 ± 0.47 | 4.52 ± 0.25 | 4.76 ± 0.37 | 0.23 |
| HCT (%) | 46.42 ± 2.50 | 45.45 ± 2.76 | 46.62 ± 2.66 | <0.001 |  | 40.43 ± 3.04 | 39.06 ± 2.87 | 41.80 ± 3.66 | 0.02 |
| MCV (fL) | 87.00 ± 6.44 | 86.19 ± 5.40 | 88.04 ± 5.99 | 0.006 |  | 86.88 ± 7.85 | 86.43 ± 5.47 | 87.85 ± 5.85 | 0.78 |
| Hemoglobin (g/dL) | 15.53 ± 0.94 | 15.45 ± 1.09 | 15.59 ± 0.94 | 0.36 |  | 13.24 ± 1.11 | 12.80 ± 1.05 | 13.73 ± 1.15 | 0.02 |
| Range: (min – max) | 117.7 – 18.1 | 10.7 – 18.3 | 13.0 – 18.5 |  |  | 10.2 – 15.6 | 11.0 – 14.6 | 11.5 – 15.7 |  |
| Full mouth bleeding scores | 3.97 ± 1.93 | 4.05 ± 1.67 | 17.77 ± 3.38 | <0.001 |  | 4.05 ± 1.73 | 5.51 ± 1.69 | 16.75 ± 3.37 | <0.001 |

Continuous variables are expressed as mean ± SD (standard deviation), and categorical variables as N [%].

Abbreviations: HDL-C, high-density lipoprotein cholesterol; LDL-C, low-density lipoprotein cholesterol; HCT, hematocrit; MCV, mean corpuscular volume.

ADDITIONAL FILE 1: TABLE S2 Multiple Logistic Regression Analyses for Localized Stage I and Stage II/III Periodontitis with Erythrocyte Indices

|  |  |  |  |  | Male |  |  |  |
| --- | --- | --- | --- | --- | --- | --- | --- | --- |
|  |  |  | Model 1 |  |  |  | Model 2 |  |
|  |  | OR | 95% CI | p-value |  | OR | 95% CI | p-value |
| Stage I | Erythrocyte (10^3^/uL) | 0.60 | 0.37 – 0.97 | 0.03 |  | 0.59 | 0.36 – 0.97 | 0.03 |
|  | HCT (%) | 0.85 | 0.79 – 0.92 | <0.01 |  | 0.85 | 0.78 – 0.91 | <0.001 |
|  | MCV (fL) | 0.98 | 0.96 – 1.01 | 0.21 |  | 0.98 | 0.96 – 1.01 | 0.21 |
|  | Hemoglobin (g/dL) | 0.88 | 0.72 – 1.08 | 0.22 |  | 0.88 | 0.72 – 1.08 | 0.22 |
|  |  |  |  |  |  |  |  |  |
| Stage II/III | Erythrocyte (10^3^/uL) | 0.77 | 0.56 – 1.06 | 0.10 |  | 0.74 | 0.53 – 1.02 | 0.066 |
|  | HCT (%) | 1.03 | 0.97 – 1.08 | 0.34 |  | 1.02 | 0.96 – 1.07 | 0.57 |
|  | MCV (fL) | 1.03 | 1.00 – 1.05 | 0.02 |  | 1.03 | 1.00 – 1.05 | 0.02 |
|  | Hemoglobin (g/dL) | 1.05 | 0.91 – 1.21 | 0.47 |  | 1.03 | 0.89 – 1.19 | 0.69 |
|  |  |  |  |  | Female |  |  |  |
|  |  |  | Model 1 |  |  |  | Model 2 |  |
|  |  | OR | 95% CI | p-value |  | OR | 95% CI | p-value |
| Stage I | Erythrocyte (10^3^/uL) | 0.21 | 0.04 – 1.30 | 0.09 |  | 0.22 | 0.04 – 1.35 | 0.10 |
|  | HCT (%) | 0.85 | 0.70 – 1.02 | 0.08 |  | 0.83 | 0.68 – 1.02 | 0.07 |
|  | MCV (fL) | 0.99 | 0.92 – 1.07 | 0.87 |  | 0.99 | 0.91 – 1.07 | 0.77 |
|  | Hemoglobin (g/dL) | 0.71 | 0.43 – 1.17 | 0.17 |  | 0.69 | 0.41 – 1.16 | 0.15 |
|  |  |  |  |  |  |  |  |  |
| Stage II/III | Erythrocyte (10^3^/uL) | 1.43 | 0.57 – 3.55 | 0.44 |  | 1.41 | 0.57 – 3.50 | 0.45 |
|  | HCT (%) | 1.15 | 0.99 – 1.32 | 0.060 |  | 1.15 | 0.99 – 1.32 | 0.06 |
|  | MCV (fL) | 1.02 | 0.96 – 1.08 | 0.58 |  | 1.02 | 0.96 – 1.08 | 0.56 |
|  | Hemoglobin (g/dL) | 1.54 | 1.00 – 2.36 | 0.04 |  | 1.52 | 1.00 – 2.32 | 0.05 |

Data are presented as odds ratios and 95% confidence intervals (CI) using multiple logistic regression analysis for

Model 1: age, educational levels, tobacco smoking, betel nut chewing and body mass index adjustments.

Model 2: age, educational levels, tobacco smoking, betel nut chewing, body mass index and leucocyte adjustments.

Abbreviations: CI, confidence interval; HCT, hematocrit; MCV, mean corpuscular volume; OR, odds ratio
